# White Esophagus: The Result of Polypharmacy

**DOI:** 10.7759/cureus.34415

**Published:** 2023-01-30

**Authors:** Brendan Andres, Pradeep R Kathi, Kai Tey

**Affiliations:** 1 Internal Medicine, University of Arizona College of Medicine - Tucson, Tucson, USA; 2 Internal Medicine/Gastroenterology, University of Arizona, Tucson, USA; 3 Gastroenterology and Hepatology, University of Arizona College of Medicine - Tucson, Tucson, USA

**Keywords:** infectious esophagitis, budesonide, immunocompetent adult, candida esophagitis, polypharmacy

## Abstract

Candida esophagitis can occur in immunocompetent patients through impairment of host defense mechanisms including salivation, esophageal motility, acidic pH, and innate immunity. Commonly prescribed medications inhibit these mechanisms, and polypharmacy has been shown to have an additive effect on promoting Candida infection. We present the case of an immunocompetent patient who was chronically prescribed multiple medications associated with Candida esophagitis but experienced infection only after the addition of oral delayed-release budesonide, which has not previously been associated with Candida esophagitis.

## Introduction

Esophageal Candida colonization has been reported in up to 12% of healthy adults [1]. Numerous medications inhibit natural defense mechanisms against Candida esophagitis. We present a case of an immunocompetent male who developed Candida esophagitis of the entire esophagus likely due to synergistic polypharmacy after the addition of oral delayed-release budesonide for the treatment of lymphocytic colitis.

## Case presentation

**Figure 1:**
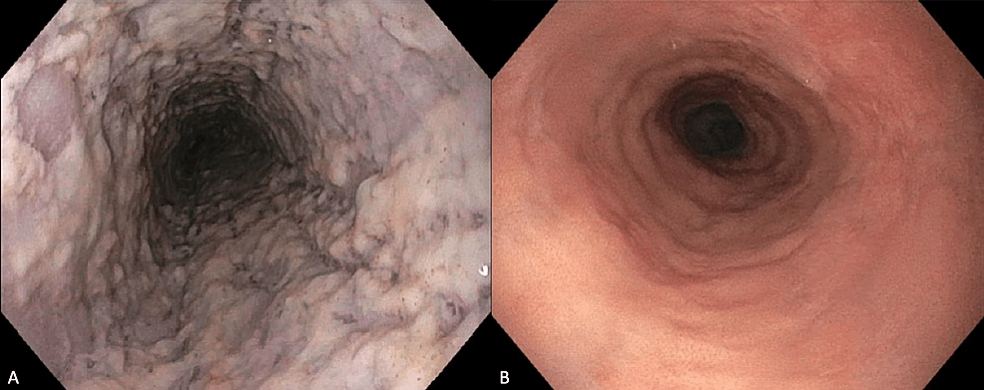
(A) Esophagogastroduodenoscopy demonstrating diffuse white plaque throughout the entire esophagus; (B) esophagogastroduodenoscopy illustrating normal esophageal mucosa after fluconazole therapy.

A 58-year-old male underwent esophagogastroduodenoscopy (EGD) for subacute odynophagia, dysphagia, and weight loss. His medical history included lymphocytic colitis, chronic gastroesophageal reflux disease, chronic obstructive pulmonary disease, asthma, and chronic neuropathy. Medications included budesonide, a proton pump inhibitor, fluticasone propionate, benralizumab, and gabapentin. The laboratory evaluation was significant for complete eosinopenia, likely due to benralizumab. A colonoscopy four months prior for diarrhea revealed lymphocytic colitis on random biopsies. Oral delayed-release budesonide 9 mg daily was subsequently prescribed for eight weeks followed by tapering which he completed two weeks prior to the initial EGD. Endoscopy showed diffuse white plaques in the entire esophagus (Figure 1A). Esophageal brushings revealed acute esophagitis with fungal yeast and pseudo-hyphae concerning for Candida esophagitis. Oral fluconazole 400 mg daily was prescribed for two weeks, which was extended by one week for persistent symptoms. A repeat EGD 12 weeks later demonstrated resolution of the white plaques and normal esophageal mucosa (Figure 1B). The biopsies were unremarkable.

## Discussion

Our case illustrates how polypharmacy can induce Candida esophagitis in an immunocompetent host through the inhibition of host defense mechanisms. Salivation, esophageal motility, acidic pH, and innate immunity together provide protection from Candida species throughout the esophagus. Proton pump inhibitors impair salivation and raise gastric pH. While proton pump inhibitors have been recognized as associated with higher rates of enteric infections, healthy patients naturally experience decreased esophageal acid exposure time owing to appropriate lower esophageal sphincter tone, thereby limiting this association with protection against Candida esophagitis of the lower esophagus [2]. Gabapentin also decreases salivation, which impairs Candida clearance via aggregation potentiated by salivary immunoglobulin A. Saliva additionally contains candicidal histatin 5 as well as candistatic calprotectin and lactoferrin proteins, which mediate the development of Candida infections [3]. Fluticasone propionate increases Candida esophagitis incidence by up to 37% in immunocompetent adults via local suppression of innate immunity [4]. Furthermore, fluticasone propionate and proton pump inhibitors synergistically increase the likelihood of developing Candida esophagitis [5]. Benralizumab depletes eosinophils through interleukin-5 receptor-mediated apoptosis, thereby interfering with the antifungal innate immunity provided by eosinophils via phagocytosis and extracellular DNA traps [6,7]. Systemic effects of oral delayed-release budesonide are reportedly limited due to a systemic bioavailability that approaches 12% in healthy patients and targeted ileal and colonic release owing to a pH-dependent coating that dissolves at pH > 5.5 and surrounding ethyl-cellulose granules [8]. Nevertheless, in the context of the patient’s chronic polypharmacy with other medications known to promote gastrointestinal Candida infections, our case suggests oral delayed-release budesonide may have additionally contributed to the development of Candida esophagitis via a synergistic mechanism given the temporal relation between its initiation and the onset of the patient’s infection. Importantly, the patient's Candida esophagitis was amenable to standard-duration anti-fungal therapy per the Infectious Diseases Society of America's guidelines [9].

## Conclusions

Candida esophagitis can occur in immunocompetent patients via synergistic inhibition of host defenses by polypharmacy. The addition of oral delayed-release of budesonide may have contributed to infection through direct mediation of innate immunity. Standard anti-fungal therapy was effective in treating this case of Candida esophagitis. Further observations are needed to confirm the additive risk of budesonide in immunocompetent patients prescribed other medications known to promote Candida esophagitis.
